# Targeted metagenomics using next generation sequencing in laboratory diagnosis of culture negative endophthalmitis

**DOI:** 10.1016/j.heliyon.2021.e06780

**Published:** 2021-04-23

**Authors:** Deepanshi Mishra, Gita Satpathy, Rohan Chawla, Daizy Paliwal, Subrat Kumar Panda

**Affiliations:** a Ocular Microbiology, Dr. R.P.Centre for Ophthalmic Sciences, All India Institute of Medical Sciences, New Delhi, India; b Department of Microbiology, All India Institute of Medical Sciences, New Delhi, India; c Vitreo Retinal Services, Dr. R.P.Centre for Ophthalmic Sciences, All India Institute of Medical Sciences, New Delhi, India; d Department of Pathology, All India Institute of Medical Sciences, New Delhi, India

**Keywords:** Metagenomics, Next generation sequencing, Endophthalmitis, Operational taxonomic units

## Abstract

To study the feasibility of 16S rRNA metagenomics using next generation sequencing (NGS) along with broad range PCR assay for 762 bp region of 16S rRNA gene with Sanger's sequencing, in microbial diagnosis of culture negative endophthalmitis. Vitreous fluid from 16 culture negative and one culture positive endophthalmitis patients, admitted to a tertiary care hospital were processed for targeted metagenomics. NGS of 7 variable regions of 16S rRNA gene was done using Ion Torrent Personal Genome Machine (PGM). Sequence data were analyzed using Ion Reporter software using QIIME and BLSATN tools and Greengenes and NCBI–Genbank databases. Bacterial genome sequences were detected in 15 culture negative and culture positive vitreous specimens. The sequence reads varied between 25,245–540,916 with read length between 142bp–228bp and coverage depth was 41.0X and 81.2X. Operational taxonomic unit (OTUs) of multiple bacterial genera and species were detected in 13 culture negative vitreous specimens and OTUs of a single bacterial species were detected in 2 culture negative and 1 culture positive specimens; one negative specimen had no bacterial DNA. Maximum numbers of OTUs detected by NGS for a bacterial species from any vitreous specimen was the one which was detected and identified by Sanger's sequencing in broad range PCR. All the bacteria were belonging to clinically relevant species. Broad range PCR with sequencing failed to identify bacteria from 5 of the 16 (31.25%) culture negative vitreous specimens. Metagenomics could detect and identify bacterial pathogens in 15 of the 16 culture negative vitreous specimen's up to species level. With rapidly decreasing cost, metagenomics has a potential to be used widely in endophthalmitis diagnosis, in which culture negativity is usually high.

## 1 Introduction

Endophthalmitis is a sight-threatening condition leading to vision loss. A significant proportion is of infectious origin (usually bacterial) which may be due to cataract surgery, intravitreal injection or trauma (Deshmukh et al., 2019). Early detection and identification of the causative organism leads to prompt and appropriate clinical management. Detection of the infective agent in endophthalmitis is a major problem. The reported culture positivity vary widely. In a prospective multi-centric study from USA the endophthalmitis vitrectomy study group (EVS) reported 69% culture positivity (Barza et al., 1997); a study from New Zealand reported 51% culture positivity (Bhikoo et al., 2020). However in countries where antibiotic use is very common and indiscriminate, most patients had already received antibiotics prior to hospital visit which may result in low culture positivity. In various studies culture positivity from endophthalmitis were reported as 29.9% (148/498) (Bhattacharjee et al., 2016), 34.6% (384/1110) (Satpathy et al., 2017), 14% (15/101) (Sharma et al., 2014), 38–44% (Deshmukh et al., 2019). In addition, culture-negativity can also be attributed to uncultivable, fastidious, slow growing organisms, or misuse of antibiotics that may inhibit microbial growth to undetectable numbers (Deshmukh et al., 2019; Durand, 2013, 2017).

Advent of molecular methods for laboratory diagnosis using PCR assays improved laboratory diagnosis by increasing sensitivity and faster detection of organisms from vitreous specimens. A major inadequacy with these assays was only pre-specified pathogens could be detected using either uniplex or multiplex PCR assay.

In the recent past broad range PCR assay have found use in diagnosis of infection and has been found to be more useful than uniplex or multiplex PCR assays targeted against specific pathogens. In the absence of clinical certainty, broad-range PCR assay using, primers targeting the 16S rRNA gene is particularly suitable as they are ubiquitous to all bacteria (Rutanga and Nyirahabimana, 2016). However in most cases broad range PCR assay fails to identify the exact bacterial species. Therefore, for appropriate antimicrobial therapy there is a need for accurate microbiological laboratory diagnosis of endophthalmitis.

Molecular methods using metagenomics and next generation sequencing has the potential to improve diagnostic yield and to provide accurate and precise laboratory diagnosis in different infections. Metagenomic 16S rRNA deep sequencing (MDS) is an unbiased high-throughput method that can detect all microorganism/pathogens present in patient's clinical specimens. This has the potential to identify known as well as unknown, novel and fastidious organisms which could not be detected earlier (Langley et al., 2015; Shea et al., 2017). Moreover polymicrobial infection can be detected as it can detect and sequence more than one DNA at one time (Kirstahler et al., 2018).

Metagenomics was used extensively in human microbiome project to detect organisms in different body sites like gut or oral cavity (Chong et al., 2012). Rapid advancement in sequencing technology and bioinformatics has made metagenomics a feasible, promising, as well as useful area in clinical diagnostics in tertiary care hospitals (Deurenberg et al., 2017; Forbes et al., 2018). Its use in clinical diagnosis particularly in culture negative infections has started in recent past (Parikh, Thermo fisher). Since culture negativity is high in endophthalmitis in countries with indiscriminate antibiotic use, it prompted us to use targeted 16S rRNA metagenomics in microbiology of endophthalmitis.

In the current study, targeted 16S rRNA metagenomics covering 7 of the 9 variable regions of 16S rRNA gene followed by next generation sequencing was undertaken for detection of bacterial pathogens in 17 vitreous fluids on Ion PGM platform along with broad range PCR assay for 762 bp region of 16S rRNA followed by Sanger's sequencing in a tertiary care eye hospital.

## 2 Materials and methods

### 2.1 Specimen collection

Vitreous specimen were collected by ophthalmologist from clinically suspected endophthalmitis patients admitted to the Dr. RP Centre for Ophthalmic Sciences, All India Institute of Medical Sciences, New Delhi, India, and undergoing vitrectomy/vitreous biopsy; 200 μL of vitreous was collected by the ophthalmologist after obtaining informed consent between April 2016 and January 2018. Ethical approval for this study was obtained from the Institute Ethics Committee of All India Institute of Medical Sciences, New Delhi-110029, (Ethical clearance Reference No.-IEC/PG/15/10/2015).

All the vitreous specimens were processed for automated culture in BD BACTEC 9050 Culture System (BD, USA) using standard protocol and 100μL vitreous specimen were kept for molecular studies. Bacterial identification was done using MALDI-TOF MS assay. Sixteen randomly selected culture negative vitreous specimens and one culture positive (*S. aureus*) specimen were further processed for 16S rRNA targeted metagenomics study.

### 2.2 Nucleic acid extraction

The total nucleic acid was extracted from above 17 vitreous specimens using commercial QIAamp DNA Mini kit (Qiagen USA). Quantity and quality of nucleic acid extracted was checked using a Nanodrop spectrophotometer 8000 (Thermo Scientific, USA). The isolated DNA was used for metagenomics deep sequencing and broad range PCR assay.

### 2.3 Metagenomics deep sequencing

16S rRNA targeted metagenomics deep sequencing was done using Ion Torrent PGM machine (USA) and Ion 16S^TM^Metagenomics kits (Life technologies, USA) as per manufactures’ instructions. With each sequencing run negative control (distilled water) was also included. Briefly:

#### 2.3.1 16S rRNA gene amplification

Multiplex PCR assays were done for amplification of 16S rRNA hypervariable regions 2, 4, and 8 in one single tube yielding amplicon fragments of ~250 bp, ~288 bp, and ~295 bp and hypervariable regions 3, 6–7, 9 in a second PCR tube yielding amplicon fragments of ~215 bp, ~260 bp, and ~209 bp respectively using primers and reagents provided with Ion 16S™ Metagenomics Kit (cat. no. A26216) (Life technologies, USA) as per manufactures’ instructions.

Equimolar quantities of PCR products were pooled and were purified with Agencourt Ampure reagent (Life technologies, USA). Fragment size and quantity of DNA were estimated as per manufactures’ instructions.

#### 2.3.2 Library preparation for deep sequencing

After end repair of the fragments, adapter/barcodes were ligated to the amplified fragments as per the instructions. A further PCR amplification of this library of amplicons was done using primers and reagents provided with the amplification kit (Life technologies, USA).

#### 2.3.3 Next generation sequencing

Next generation sequencing of the amplified library was done after a round of emulsion PCR using Ion PGM™ Hi^TM^Q™ OT2 Kit™ 400 (Life technologies, USA) and sequencing was done using Ion PGM™ Hi^TM^Q™ sequencing reagents on a 318 (1000M.b.p.) micro-chip as per manufacturers’ instructions.

#### 2.3.4 Sequence analysis

Post sequencing base calling and adaptor trimming was performed using the computer programme Torrent Suite (Life technologies, USA). The output reads were aligned and mapped using Ion Reporter™ software v5.10 (USA) with default parameters (Thermo Fisher Scientific) for metagenome analysis including read mapping, annotation and reporting. 16S rRNA was analyzed with the QIIME suite software tools (v1.8). The filtered sequence reads (Phred ≥ Q20) were used to pick the operational taxonomic units (OTUs), with an open-reference OTU picking method based on 97% identity to entries in the Greengenes database (v13.5) as per manufacturer's instructions.

Further, FASTQ files were processed for blast analysis for homology against available genes sequences in Genbank database using NCBI blast, computer programme (http://www.mibi.nim.nih.gov). The sequencing data was uploaded to Ion reporter database (https://ionreporter.thermofisher.com/ir/) and to Genbank and accession numbers were obtained.

### 2.4 Broad range PCR assay

Broad range PCR assay was performed in the isolated DNA for amplification of 762 bp of 16S rRNA gene using published primers (Xu et al., 2004) using standard strain of *E.coli* (ATCC 25922) as a positive control and sterile distilled water as a negative control. The PCR assays were done using the standardized parameters in a thermal cycler (Applied Bio system, USA) as described earlier (Mishra et al., 2018). Amplicons were electrophoresed on 1.5% agarose gel and visualized under a Gel documentation system (UVP, USA). The amplified DNA fragments were purified from the gel using QIAquick Gel Extraction Kit (QIAgen, USA) as per the manufacturers’ instructions.

Nucleotide sequencing was done directly on an ABI Prism 310 genetic analyzer (Applied Biosystem, Foster City, CA) as was discussed earlier (Mishra et al., 2018). The nucleotide sequences were aligned using DNASTAR laser gene molecular biology suite software and aligned sequences were analyzed for homology in the Gene Bank database using NCBI BLAST computer programme (http://www.mibi.nim.nih.gov.). The nucleotide sequences of microorganisms determined as per CLSI MM18A document guidelines were deposited and accession numbers were obtained from NCBI databank (Mishra et al., 2018).

## 3 Results

From the 17 endophthalmitis patients, 8 were of post surgical origin, 5 were of post traumatic, one was post avastin injection and in 3 aetiology were not known. One of the vitreous specimen (post traumatic) had low DNA yield after DNA isolation, so only 16 were further processed.

**Figure 1:**
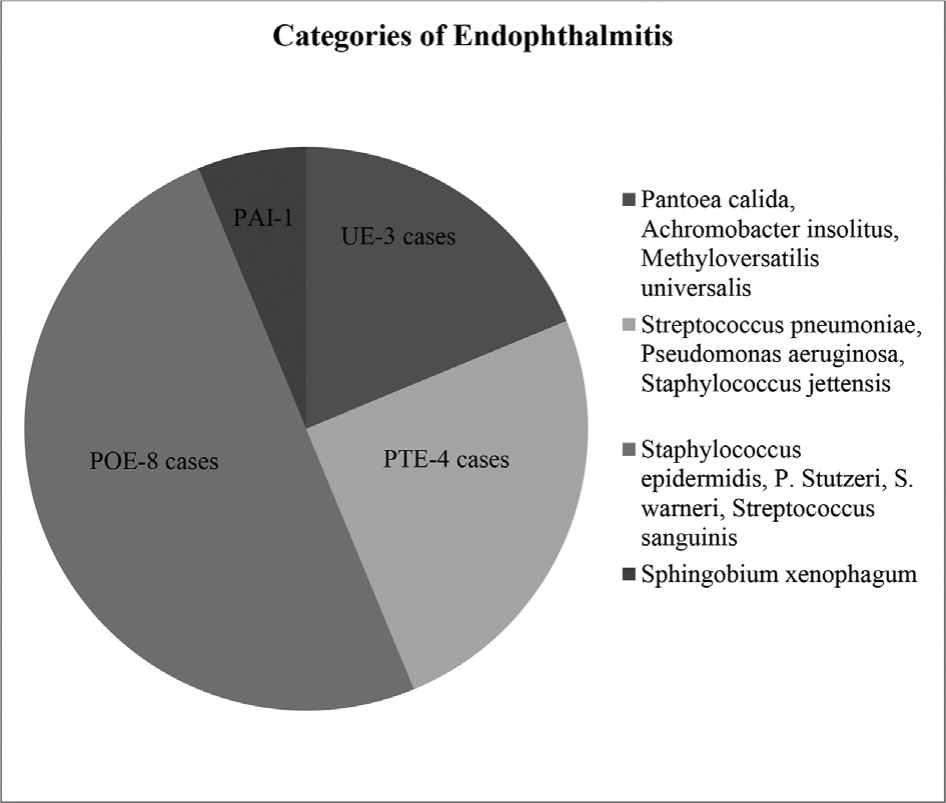
Krona chart showing bacterial species detected and identified with highest OTUs among different categories of endophthalmitis (PAI∗- Post avastin injection, POE∗- Post operative endophthalmitis, PTE∗- Post traumatic endophthalmitis, UE∗- Unknown etiology).

*Metagenomics deep sequencing*: By next generation sequencing in all 16 vitreous specimens, the number of bases obtained varied from 3,339,355 to 93,284,496 at Phred ≥ Q20. The average read length varied from 142bp-228bp. The number of reads obtained varied from 25,245 to 540,916 (Table 1). The Krona chart (Figure 1) visualized by consensus is given.

**Table 1:** Next generation sequencing run information for vitreous specimens.

S. No.	No. of reads	No. of bases Q20	Average read length	Average coverage depth
1	1,23,661	1,45,66,785	142bp	41.0X (Run 2)
2	45,315	66,14,097	176bp	41.0X (Run 2)
3	76,476	1,12,46,790	177bp	41.0X (Run 2)
4	52,584	79,76,995	184bp	41.0X (Run 2)
5	83,971	1,27,55,632	184bp	41.0X (Run 2)
6	3,63,086	5,41,44,247	179bp	41.0X (Run 2)
7	71,833	1,20,33,565	197bp	41.0X (Run 2)
8	25,245	33,39,355	159bp	41.0X (Run 2)
9	1,58,096	2,28,36,683	170bp	81.2X (Run 1)
10	5,40,916	8,47,69,107	182bp	81.2X (Run 1)
11	2,34,621	4,03,10,651	190bp	41.0X (Run 2)
12	4,24,799	8,60,60,812	221bp	41.0X (Run 2)
13	2,74,666	5,75,01,500	228bp	41.0X (Run 2)
14	4,76,456	9,32,84,496	213bp	41.0X (Run 2)
15	4,76,571	9,24,56,779	216bp	81.2X (Run 1)
16	2,53,648	4,88,47,828	212bp	81.2X (Run 1)

Q20- Phred score.

In none of the specimens singleton OTUs was obtained. Only in 3 vitreous specimens (one culture positive and 2 culture negative), OTUs of a single bacterium (varying from 238 to 3190) were obtained. In rest 13 culture negative specimens OTUs belonging to multiple bacteria were obtained. The bacteria could be identified up to species level from each clinical specimen. Detailed result of bacteria detected and identified is given in Table 2 with the number of OTUs present and genbank accession numbers.

**Table 2:** Bacteria detected and identified using broad range PCR assay and next generation sequencing from vitreous specimens with their accession numbers.

S. No.	Broad range 16S rRNA PCR assay/Sanger sequencing	Results of next generation sequencing (OTU reads Genus level)	Results of next generation sequencing (OTU reads species level)	Accession number
1 (UE)	No significant match found	Pantoea 5934, Paracoccus 60, Acinetobacter 59, Erwinia 23	*Pantoea calida* 3900, *Erwinia dispersa* 13	MN049835-MN049837
2 (PTE)	*S. pneumoniae*	Streptococcus 9128	*Streptococcus pneumoniae* 713, *Streptococcus infantis* 21	MN049856, MN049857
3 (PTE)	*S. aureus*	Staphylococcus 14228	*Staphylococcus aureus* 3190	MN049840, MN049841
4 (POE)	*S. epidermidis*	Staphylococcus 10000	*Staphylococcus epidermidis* 238	MN049858, MN049859
5 (POE)	*S. epidermidis*	Staphylococcus 14982	*Staphylococcus epidermidis* 962, *Staphylococcus auricularis* 629	MN049883, MN049884
6 (PTE)	*P. aeruginosa*	Pseudomonas 57537, Acinetobacter 17	*Pseudomonas aeruginosa* 17602, *Pseudomonas mendocina* 247, *Pseudomonas sp.* 53, *Pseudomonas alcaligenes* 31, *Pseudomonas indica* 13	MN049828, MN049829
7 (PTE)	*Uncultured bacterium*	Staphylococcus 17306, Sphingomonas 142, Novosphingobium 24, Rhizobium 11, Micrococcus 11	*Staphylococcus jettensis* 8922, *Staphylococcus aureus* 3276, *Staphylococcus haemolyticus* 2066	MN049842-MN049845
8 (POE)	*S. epidermidis*	Staphylococcus 1575, Acinetobacter 33, Roseomonas 12, Methylobacterium 12, Nocardia 10	*Staphylococcus epidermidis* 1575	MN049869- MN049874
9 (PAI)	No significant match found	Sphingobium 19, Aerococcus 10	*Sphingobium xenophagum* 19, *Aerococcus urinaeequi* 10	MN049854, MN049855
10 (UE)	No significant match found	Achromobacter 67067	*Achromobacter insolitus* 22135, *Achromobacter denitrificans* 24, *Achromobacter sp.* 14, *Achromobacter xylosoxidans* 11	MN049838, MN049839
11 (POE)	*P. Stutzeri*	Pseudomonas 26482, Acinetobacter 4899, Rubellimicrobium 4101, Paracoccus 4760, Paracoccus 4760 Anabaena 3474, Serinicoccus 2126, Nocardioides 1906, Streptococcus 1331,Exiguobacterium 1077, Sphingomonas 966, Corynebacterium 973, Ornithinimicrobium 918, Rhizobium 878	*P. Stutzeri* (1452)*, Rubellimicrobium roseum* (1451)*, Acinetobacter junii* (1028)	MN049875-MN049880
12 (POE)	*S. warneri*	Staphylococcus 229312	*S. warneri* (105156), *S. pasteuri* (31188), *S. jettensis* (1190), *S. epidermidis* (695), *S. cohnii* (371)	MN049881, MN049882
13 (POE)	*S. epidermidis*	Staphylococcus (131580), Acinetobacter (1444), Cell vibrio 302 Enhydrobacter (248), Propionibacterium (177), Peredibacter (97), Paracoccus (92), Corneybacterium (92)	*S. epidermidis* (11771), *S. hominis* (872), *Enhydrobacter aerosaccus* (248), *Propionibacterium acnes* (177), *Cellvibrio gangdavensis* (156), *Peredibacter starrii* (97), *S. auricularis* (79), *S. aureus* (56)	MN049830-MN049834
14 (POE)	*S.epidermidis*	Staphylococcus 193407, Acinetobacter 12474, Pseudomonas 1750, Flavobacterium 1274, Sphingomonas 913, Cellvibrio 499, Legionella 454, Propionibacterium 408, Corynebacterium 185, Bacteroides 344, Aeromonas 389	*Staphylococcus epidermidis* 4287, *Acinetobacter lwoffii*3079, *Acinetobacter towneri* 2028, *Staphylococcus hominis*700, *Propionibacterium acnes* 408, *Sphingomonas dokdonensis* 320, *Acinetobacter schindleri* 309, *Pseudomonas lini* 209	MN049846- MN049853
15 (UE)	No significant match found	Methyloversatilis 20168, Acinetobacter 18329, Pseudomonas 10370, Corneybacterium 9161, Moraxella 7507, Staphylococcus 8312, Propionibacterium 6346, Hydrogenophaga 3429, Malikia 2462, Brevundimonas 2315, Methylobacterium 1586, Sphingobium 1586, Burkholderia 1232, Nocardioides 1177, Sphingomonas 1079,	*Methyloversatilis universalis* (19650)*, P. acne* (6113)*, Acinetobacter baylyi* (4969)*, Acinetobacter sp.* (2965)*, Corynebacterium halotolerans* (2235)	MN049860- MN049868
16 (POE)	No significant match found	Streptococcus 98424, Acinetobacter 819, Paracoccus 136, Cellvibrio 130, Aeromonas 116	*Streptococcus sanguinis* 45387*, Cellvibrio gandavensis* 113*, Enhydrobacter aerosaccus* 90*, Acinetobacter baylyi* 81*, Legionella lytica* 51*, Streptococcus lactarius* 31, *Streptococcus minor* 25, *Streptococcus troglodytidis* 17	MN049825-MN049827

POE∗- Post operative endophthalmitis, PTE∗- Post traumatic endophthalmitis, PAI∗- Post avastin injection, UE∗- Unknown etiology.

The bacteria present in the vitreous specimen with highest number of OTUs were as follows; *Staphylococcus epidermidis* was the commonest bacteria, detected in 5 of the 8 post operative endophthalmitis, in rest 3 *Staphylococcus warneri, Pseudomonas stutzeri and Streptococcus sanguinis* were detected. The bacteria detected in 4 post traumatic endophthalmitis cases were *Staphylococcus jettensis*, *P. aeruginosa, S. pneumoniae and S. aureus*. In the post avastin injection specimen *Sphingobium xenophagum* was detected. *Pantoea calida, Achromobacter insolitus and Methyloversatilis universalis* were detected in 3 cases of endophthalmitis of unknown etiology respectively.

*Broad range PCR assay:* This was positive in 16 patients including the culture positive one. The bacteria could not be identified from the nucleotide sequence from 5 of these 16 specimens. The identified bacteria (with highest sequence match with available bacterial sequences in Gene Bank data base) and unidentified bacteria are given in Table 2. The identified bacteria were the same for which highest numbers of OTUs were detected by NGS.

## 4 Discussion

Early and accurate laboratory diagnosis leading to appropriate treatment can be vision saving in endophthalmitis. Clinical metagenomics is an evolving field, which has proven promising in diagnosis of different infections. High throughput sequencing such as next generation sequencing (NGS) has simplified the metagenomics technique and made it feasible for use in clinical microbiology laboratory (Doan et al., 2016, 2017). In clinical metagenomics, a widely used method is targeted metagenomics, in which amplification and deep sequencing 16S rRNA gene of bacteria is done and the sequence data is analyzed for detection and identification of organisms. This is more feasible than whole genome sequencing in routine clinical laboratories (Doan et al., 2016, 2017).

In the current study we have used 16S metagenomics using next generation sequencing on Ion Torrent PGM platform (Ion Torrent, USA) for 7 variable regions of 16S rRNA. For getting best results with highest resolution, ideally the entire 16S rRNA gene region needs sequencing. Since this is not possible in clinical settings, the variable regions present in the 16S rRNA gene (total 9) are to be sequenced, the more numbers of variable regions sequenced the better the result (Watanabe et al., 2018; Bukin et al., 2019). We have deep sequenced 7 of the 9 variable regions of 16S rRNA to get accurate results. Previous studies using different variable regions have shown that, these 7 variable regions had given the best results for metagenomics (Bukin et al., 2019; Ion Torrent application note). In most other studies involving clinical specimens, lesser number of variable regions were used with variable results. In one, amplification of V3–V4 regions 16SrRNA was used separately for determining the composition of gut microbiota, and discrepant results were obtained (Milani et al., 2016). The results for these two regions differed from each other and no conclusion could be drawn (Milani et al., 2016). In another study V3–V4 regions of 16SrRNA were used to detect bacteria from culture negative endophthalmitis and this generated reproducible sequence reads up to the genus level but not up to the species level. So only the genera of bacteria present could be known, not the exact bacterial species (Deshmukh et al., 2019). In a study two primer sets for V1/V2 and V3/V4 region were used for bacterial detection from female genital tract. Primer set for V1/V2 region failed to detect bacteria of importance in vagina while V3/V4 hypervariable region gave a better result (Graspeuntner et al., 2018).

Highest numbers of OTU reads detected in NGS were for the bacteria detected by broad range PCR assay in majority of the specimens. These 16 vitreous specimens were also subjected to broad range PCR assay for 16S rRNA gene, as it is not selective for any particular bacteria. In this study by sequencing the 7 variable regions of 16S rRNA gene, bacteria could be detected in 16 vitreous specimens (15 culture negative and 16 broad range PCR positive) up to family, genus and species level in each specimen. In 6 of these 16 broad range PCR positive and culture negative vitreous specimens the identity of the bacterial species could not be determined (1 of these 6 was determined as uncultured bacterium) from sanger sequencing. However, these 6 bacteria could be identified using next generation sequencing results in this study. These bacterial species were: *Pantoea calida*, *Sphingobium xenophagum, Achromobacter insolitus, Methyloversatilis universalis, Streptococcus sanguinis and Staphylococcus jettensis* (determined as uncultured bacterium). This was possible because of the increased resolving power using 7 variable regions (Kirstahler et al., 2018; Torrent application note). Therefore all the bacteria detected could be exactly identified irrespective of the presence of single species or multiple species. Multiple species were present in 13 specimens.

NGS could provide quantitative data in the form of number of sequence reads which were binned in to the bacterial OTUs by computer software analysis (Ion Reporter and QIIME). It has been suggested that a minimum of 10,000–15,000 reads in a specimen gives almost accurate results (Bukin et al., 2019). In our study the read numbers were much higher with a minimum of 25,245 in specimen number 8. In 13 of the 16 specimens OTUs of varying numbers for multiple bacteria were present. However, number of OTUs of one bacterium always far exceeded the number of OTUs for other bacteria.

Other studies have interpreted the presence of OTUs for multiple bacteria as suspected poly microbial infections in endophthalmitis (Deshmukh et al., 2019; Kirstahler et al., 2018). In our study in most instances the number of OTUs of one bacterium was least several logs higher than those for other bacteria; eg. 3900 OTUs of *Pantoea calida* and 13 OTUs of *Erwinia dispersa* were present in specimen number one. In our study, in 8 of the 13 specimens, the number of OTUs of one bacterial species were detected in numbers at least 3 logs higher than those of others. In rest 5 they were present in significantly larger numbers. Therefore, we propose that *Pantoea calida* might have been the current causative agent, whereas *Erwinia dispersa* DNA sequence might have been present due to some past infection or leakage from some other sites which were present in the body as circulating DNAs. Since this field is new, the exact cut off difference between the numbers of OTUs to call it present or past infection are yet to be fixed and to know which is the exact current infective agent. This may require larger studies.

In a study in which multiple bacteria were detected in clinical specimens using metagenomics, these could be identified in 58.2% instances up to species level and in 74.5% instances up to genus level due to availability of only incomplete or variably curated databases (Forbes et al., 2018). In yet another study NGS could detect multiple bacteria in most of the culture-negative and few culture positive vitreous specimens up to genus level only (Deshmukh et al., 2019). In the current study, all the bacteria present were identified up to species level. They opined that detection of multiple organisms in specimens were due to presence of DNA of several viable or non-viable microbes which might have been present before.

The presence of bacterial sequences in our culture negative endophthalmitis might have been due to prior antibiotic therapy or presence of uncultivable or fastidious/slow growing organisms as was suggested earlier (Deshmukh et al., 2019; Kirstahler et al., 2018).

In the present study potentially pathogenic bacteria were detected, in both culture negative as well as culture positive specimens. Rarely reported organisms like: *Pantoea calida*, *Sphingobium xenophagum, Achromobacter insolitus, Methyloversatilis universalis and Streptococcus sanguinis* were detected in 5 culture negative specimens. All of these bacteria have been associated with human infections earlier, as described below (Forbes et al., 2018).

*Pantoea spp.* a gram-negative bacilli, was reported from aqueous humour from Korea in 2010 (Lee et al., 2010), and from blood of a 77-year-old woman with end-stage stomach cancer in 2010 (Yamada et al., 2017). *Sphingobium xenophagum and Methyloversatilis universalis* were not reported earlier from endophthalmitis. However these were detected from ocular microbiome of contact lens wearer in 2016 by deep sequencing (Shin et al., 2016). *Achromobacter insolitus* has been reported from endophthalmitis by culture and molecular methods (Coenye et al., 2003). *Streptococcus sanguinis* was earlier reported from endophthalmitis (Greenfield et al., 1996). *Staphylococcus jettensis* although has been reported from other infections (Becker et al., 2014) has not yet been reported from endophthalmitis. *S. epidermidis* (5/16) was the single most common bacterium detected from vitreous specimen in this study, which corroborates the results using culture and molecular methods (Mishra et al., 2018; Shin et al., 2016).

In conclusion, using the next generation sequencing data, the bacteria which had the presence of highest numbers of OTUs in a specimen was considered as the most probable causative bacterial pathogen in the current episode of endophthalmitis. Targeted metagenomics and next generation sequencing on Ion PGM platform was found to be a feasible technology for providing rapid and accurate microbial detection giving microbial diagnosis up to species level. Currently it is expensive, requires special skill and infrastructure and the results need careful interpretation. With rapidly decreasing cost, metagenomics has a potential to be used widely in endophthalmitis.

## Declarations

### Author contribution statement

Deepanshi Mishra: Performed the experiments; Analyzed and interpreted the data; Wrote the paper.

Gita Satpathy, Subrat Kumar Panda: Conceived and designed the experiments; Analyzed and interpreted the data; Contributed reagents, materials, analysis tools or data; Wrote the paper.

Rohan Chawla: Contributed reagents, materials, analysis tools or data.

Daizy Paliwal: Performed the experiments.

### Funding statement

Deepanshi Mishra was supported by Internal funding from All India Institute of Medical Sciences.

### Data availability statement

Data included in article/supplementary material/referenced in article.

### Declaration of interests statement

The authors declare no conflict of interest.

### Additional information

No additional information is available for this paper.
